# Different screening strategies (single or dual) for the diagnosis of suspected latent tuberculosis: a cost effectiveness analysis

**DOI:** 10.1186/1471-2466-10-7

**Published:** 2010-02-22

**Authors:** Anil Pooran, Helen Booth, Robert F Miller, Geoff Scott, Motasim Badri, Jim F Huggett, Graham Rook, Alimuddin Zumla, Keertan Dheda

**Affiliations:** 1 Centre for Infectious Diseases and International Health, Division of Infection and Immunity, University College London Medical School, University College London, 43 Cleveland Street, London, W1T 4JF, UK; 2 TB Clinic, University College London Hospitals NHS Trust, London, WC1E 6AU, UK; 3 Department of Population Sciences and Primary Care, University College London Medical School, University College London, London, WC1E 6JB, UK; 4 Division of Pulmonology and UCT Lung Institute, Department of Medicine, University of Cape Town & Groote Schuur Hospital, Observatory Cape Town, 7925, South Africa; 5 Institute of Infectious Diseases and Molecular Medicine, University of Cape Town, South Africa

## Abstract

**Background:**

Previous health economic studies recommend either a dual screening strategy [tuberculin skin test (TST) followed by interferon-γ-release assay (IGRA)] or a single one [IGRA only] for latent tuberculosis infection (LTBI), the former largely based on claims that it is more cost-effective. We sought to examine that conclusion through the use of a model that accounts for the additional costs of adverse drug reactions and directly compares two commercially available versions of the IGRA: the Quantiferon-TB-Gold-In-Tube (QFT-GIT) and T-SPOT.*TB*.

**Methods:**

A LTBI screening model directed at screening contacts was used to perform a cost-effectiveness analysis, from a UK healthcare perspective, taking into account the risk of isoniazid-related hepatotoxicity and post-exposure TB (2 years post contact) using the TST, QFT-GIT and T-SPOT.*TB *IGRAs.

**Results:**

Examining costs alone, the TST/IGRA dual screening strategies (TST/T-SPOT.*TB *and TST/QFT-GIT; £162,387 and £157,048 per 1000 contacts, respectively) cost less than their single strategy counterparts (T-SPOT.*TB *and QFT-GIT; £203,983 and £202,921 per 1000 contacts) which have higher IGRA test costs and greater numbers of persons undergoing LTBI treatment. However, IGRA alone strategies direct healthcare interventions and costs more accurately to those that are truly infected.

Subsequently, less contacts need to be treated to prevent an active case of TB (T-SPOT.*TB *and QFT-GIT; 61.7 and 69.7 contacts) in IGRA alone strategies. IGRA single strategies also prevent more cases of post-exposure TB. However, this greater effectiveness does not outweigh the lower incremental costs associated with the dual strategies. Consequently, when these costs are combined with effectiveness, the IGRA dual strategies are more cost-effective than their single strategy counterparts. Comparing between the IGRAs, T-SPOT.*TB*-based strategies (single and dual; £39,712 and £37,206 per active TB case prevented, respectively) were more cost-effective than the QFT-GIT-based strategies (single and dual; £42,051 and £37,699 per active TB case prevented, respectively). Using the TST alone was the least cost-effective (£47,840 per active TB case prevented). Cost effectiveness values were sensitive to changes in LTBI prevalence, IGRA test sensitivities/specificities and IGRA test costs.

**Conclusion:**

A dual strategy is more cost effective than a single strategy but this conclusion is sensitive to screening test assumptions and LTBI prevalence.

## Background

Identification and treatment of latent tuberculosis cases remains an effective strategy in the control of tuberculosis (TB). The tuberculin skin test (TST) has been the primary tool for identifying these individuals. However, the recently introduced interferon-gamma release assays (IGRAs), which measure IFN-γ responses to the relatively TB-specific antigens ESAT-6 and CFP-10, have been gaining acceptance in the past few years as potential replacements for the TST. In low burden settings IGRAs correlate better with exposure to *M. tuberculosis *than TST (reviewed in [1-3]) and are more specific in BCG-vaccinated subjects [4], particularly when BCG is given after infancy [5].

Two versions of the IGRA are now commercially available: The T-SPOT^®^.*TB *(Oxford Immunotec Ltd, Oxford, UK) and Quantiferon^®^-TB Gold In-tube (Cellestis Ltd., Carnegie, Australia), which use an ELISPOT and ELISA format, respectively. While both have improved performance characteristics over the TST in low burden settings, T-SPOT.*TB *may be more sensitive and associated with less indeterminate results, especially in an immunocompromised population [2,6].

Varying guidelines on the application of these assays have been published [7,8]. The Centers for Disease Control and Prevention, USA, recommends that IGRA can replace TST in all settings [8]. By contrast, in the UK, the National Institute of Health and Clinical Excellence (NICE) recommends the use of IGRAs using a dual testing strategy, where IGRA is only performed on individuals who have a positive TST result [7]. Several other countries have adopted a similar approach (e.g. France, Canada). These considerations are based mainly on cost effectiveness calculations. We sought to re-examine this choice as the NICE analysis [7] was based on only limited data about the IGRAs. Since then only a limited number of independent cost-analyses have been performed [9-16] and none have directly compared the cost effectiveness of screening with both standardized versions of the IGRAs. Furthermore, none of these subsequent studies have been performed in the UK, where healthcare costs may be different to those in other European countries, costs related to post-exposure TB were not included in some models [13,14], and only half of these studies [11,15,16] take into account the effect of isoniazid-induced hepatotoxicity. Whilst uncommon (~0.1% to 0.6%), this adverse drug-reaction may be costly to treat and even fatal [17].

In this study, a decision tree was constructed to measure the costs and clinical outcomes (i.e. effectiveness) over a 2 year period of screening a cohort of 1,000 individuals, from a UK healthcare perspective, using either a single (TST or IGRA) or dual (TST followed by IGRA) strategy. Both versions of the IGRA (T-SPOT.*TB *and QFT-GIT) were evaluated in the analysis. The base case of the analysis was chosen to represent a cohort of close contacts of infectious TB cases, but the variation of the parameters in the sensitivity analysis allows some conclusions to be drawn about cohorts with differing epidemiology.

## Methods

### Model Structure

**Figure 1:**
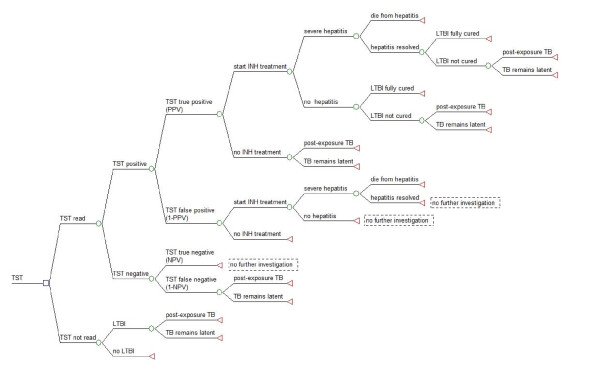
**TST screening strategy for diagnosis of presumed latent TB infection (LTBI)**. A decision tree for the diagnosis of LTBI using the TST alone in a single test strategy. Square nodes represent decision branches, circular nodes represent chance branches and triangular nodes represent terminal branches.

**Figure 2:**
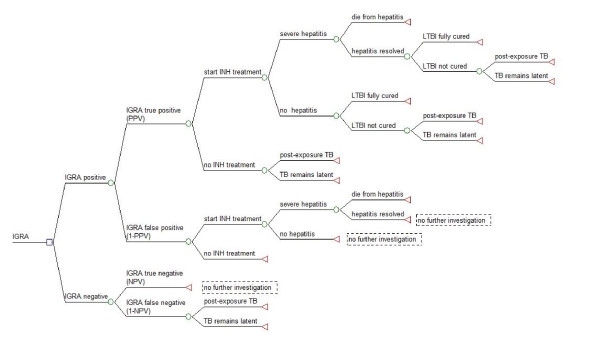
**IGRA (T-SPOT.*TB or *QFT-GIT) screening strategy for diagnosis of presumed latent TB infection (LTBI)**. A decision tree for the diagnosis of LTBI using the IGRA (T-SPOT.*TB *or QFT-GIT) alone in a single test strategy. Square nodes represent decision branches, circular nodes represent chance branches and triangular nodes represent terminal branches. The same decision tree was used for both versions of IGRA single strategies as they both have identical screening steps in each scenario.

**Figure 3:**
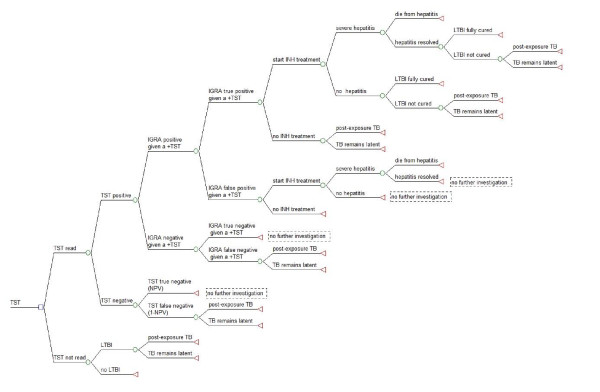
**Dual screening strategy (IGRA on all TST positive) for diagnosis of presumed latent TB infection (LTBI)**. Decision tree for the diagnosis of LTBI using a dual diagnostic strategy (TST in all cases followed by the T-SPOT.*TB *or QFT-GIT for a positive TST result). Square nodes represent decision branches, circular nodes represent chance branches and triangular nodes represent terminal branches. The same decision tree was used for both versions of the IGRA dual strategy as they both have identical screening steps in each scenario.

A decision tree was used to represent the clinical pathways associated with screening close contacts of infectious TB index cases. Five different screening scenarios were investigated in this cost-effectiveness analysis: (1) TST alone, (2) the T-SPOT.*TB *assay alone, (3) TST followed by T-SPOT.*TB *assay when TST was positive (TST/T-SPOT.*TB*), (4) Quantiferon-TB-Gold-In-Tube (QFT-GIT) alone, and (5) TST followed by QFT-GIT when TST was positive (TST/QFT-GIT). Construction of the decision tree and analysis was performed using TreeAge Pro 2009 (TreeAge Software Inc., Williamston, MA, USA) and Microsoft Excel 2003 (Microsoft, USA). Decision trees are shown in figures 1, 2 and 3. See additional file 1: supplemental data for a description of the decision tree models. No ethical approval was required for this study.

### Model Parameters

Probability values were sourced from published literature. Cost estimates were taken from UK national sources or from published literature when data from these sources were unavailable. All costs were updated to 2008 GBP using the Bank of England Consumer Price Index [18]. There was no time discounting of future costs as the time period of the model was only 2 years. All costs and probabilities are shown in table 1. See additional file 1: supplemental data for details of the model parameters.

**Table 1:** Probabilities and cost-estimates used in the cost-analysis for screening of LTBI.

Variable	Baseline	Range	Source
**Probabilities**			

Prevalence of LTBI	0.30	0.1-0.4	[24,39,40]

TST return rate	0.90	0.65-0.95	[14,27]

T-SPOT.*TB *sensitivity	0.95	0.83-0.97	[30,41-43]

T-SPOT.*TB *specificity	1.00	0.92-0.99	[41,43]

T-SPOT.*TB *positive result	0.285		Calculated

T-SPOT.*TB *true positive (PPV)	1.000		Calculated

T-SPOT.*TB *true negative (NPV)	0.979		Calculated

T-SPOT.*TB *true positive (PPV) given a positive TST	1.000		Calculated

T-SPOT.*TB *negative (NPV) given a positive TST	0.987		Calculated

QFT-GIT sensitivity	0.89	0.85-0.95	[32,44]

QFT-GIT specificity	0.95	0.90-0.97	[32,44]

QFT-GIT positive result	0.302		Calculated

QFT-GIT true positive (PPV)	0.884		Calculated

QFT-GIT true negative (NPV)	0.953		Calculated

QFT-GIT true positive (PPV) given a positive TST	0.930		Calculated

QFT-GIT true negative (NPV) given a positive TST	0.971		Calculated

TST sensitivity	0.85	0.69-0.95	[30,41,42,45]

TST specificity	0.80	0.65-0.90	[20,46]

TST positive result	0.395		Calculated

TST true positive (PPV)	0.646		Calculated

TST true negative (NPV)	0.926		Calculated

Start INH treatment	0.80	0.55-0.95	[47]

Develop severe INH hepatitis	0.003	0.003-0.041	[47-50]

Death due to hepatitis	0.00002	0.00001-0.0001	[51,52]

Efficacy of 3 months INH (LTBI fully cured)	0.21	0.1-0.3	[23,24,53]

LTBI not cured (with 3 months INH)	0.79	0.7-0.9	[23,24,53]

Efficacy of 6 months INH (LTBI fully cured)	0.65	0.5-0.93	[10,17,23,49,53,54]

LTBI not cured (with 6 months INH)	0.35	0.07-0.5	[10,17,23,49,53]

Post exposure TB	0.025	0.01-0.05	[14,55]

TB remains latent	0.975	0.95-0.99	[14,55]

**Cost (British Pounds; £s)**			

T-SPOT.*TB *(kit, consumables and processing) + phlebotomy	55.00	45.00-100.00	Test cost from Royal Free Hospital, London; Phlebotomy cost from [56]

TST(cost of disposables, administration and reading)	16.14	8.07-32.28	[7,18,57]

QFT-GIT (kit, consumables and processing) + phlebotomy	45.00	35.00-80.00	Test cost from Royal Blackburn Hospital; Phlebotomy cost from [56]

Treatment for severe INH hepatotoxicity	629.12	314.56-1258.24	[18,20,56]

3 months INH treatment	484.38	242.19-968.76	Calculated from [7,18]

6 months INH treatment	524.59	262.30-1049.18	[7,18]

Treatment for active TB	7619.67	3809.84-15239.34	[18,37]

(LTBI-latent tuberculosis infection, TST-Tuberculin Skin Test, INH-isoniazid, PPV-positive predictive value, NPV-negative predictive value).

### Effectiveness Measures

The number of active TB cases prevented and the number-needed-to treat (NNT) i.e. the number of people treated for LTBI to prevent an active TB case was calculated for each strategy. Cost-effectiveness was measured as the total cost per active TB case prevented and the incremental cost per active TB case prevented. See additional file 1: supplemental data for details on how these measures were calculated.

### Model Assumptions

(i) All contacts who may already have active TB would be symptomatic and be identified at initial clinical examination (approximately 1% of contacts have active TB on initial screening [19]). We acknowledge the possibility that a negligible proportion of contacts may have active disease, but be asymptomatic, and would therefore be missed at the time of initial examination. Therefore, we assume there are no active cases at the time of testing. (ii) Close contact with an infectious TB case in association with laboratory evidence of latent tuberculosis infection (LTBI) is an indication for LTBI preventative treatment in the UK. Therefore, it is assumed that the test (either IGRA or TST) result is the only indicator for LTBI and treatment is given solely based on this assumption. (iii) As there is no gold-standard for LTBI detection, test sensitivities and specificities were obtained from confirmed TB cases and healthy contacts at low risk for exposure, respectively (iv) We assume that isoniazid treatment will cease if severe drug-induced hepatitis develops, while mild rises in transaminase levels would result in continued usage with monitoring of liver function tests, or substitution with another drug (ethambutol or rifampicin) at no extra cost [19-21]. We assumed that hepatitis would develop in the first three months of treatment [22,23]. In order to keep the model simple, we assigned a treatment stop-point of 3 months, as assumed in other cost analyses [11,15]. Therefore, a person developing severe hepatitis will only receive a 3 month course of isoniazid, which provides only partial protection. Conversely, a person who does not develop hepatitis will receive a full 6 months isoniazid (recommended in the UK in contrast to 9 months in the USA) and will benefit from better protection [24]. (v) The model only examines post-exposure cases of TB occurring within a 2 year period, and also does not take into account further spread of TB into the population. It also assumes that following chemoprevention, individuals do not become re-infected.

## Results

### Base case analysis

Cost and probability estimates were inputted into the decision tree model to determine associated costs and effectiveness measures of each screening strategy. Total and component costs for the base case are shown in table 2. In costs terms alone, the two IGRA single screening strategies were the most expensive, with the T-SPOT.*TB *and the QFT-GIT almost identical in overall cost at £203,983 and £202,921 per 1000 contacts screened, respectively. Test costs comprised a significant proportion (~25%) of the total costs of these two strategies. Conversely, the TST/QFT-GIT and TST/T-SPOT.*TB *dual strategies were the least costly at £157,048 and £162,387 per 1000 contacts screened, respectively. However, the breakdown of where those costs go is revealing. The dual strategies had higher costs resulting from false negative results due to the combination of false negative TST and IGRA results. The TST alone strategy (£199,589 per 1000 contacts) had the lowest test costs (£15,433) but this strategy incurred the highest costs resulting from test inaccuracies (£70,081), particularly the costs incurred on false positive results (£55,090). If no screening is performed, then this still incurs downstream costs due to the treatment of resulting active TB cases; this amounts to £57,148 per 1000 contacts over the 2 year period of the model.

**Table 2:** Analysis of costs of each screening strategy

Cost Measures	TST	T-SPOT.TB	QFT-GIT	TST/T-SPOT.TB	TST/QFT-GIT	No screening
Treatment & follow-up costs	£184,156	£148,983	£157,921	£127,402	£125,618	£57,148

Test costs	£15,433	£55,000	£45,000	£34,986	£31,431	£0

**Total costs**	**£199,589**	**£203,983**	**£202,921**	**£162,387**	**£157,048**	**£57,148**

**Breakdown of costs associated with test inaccuracies**						
Costs incurred on false positives	£55,090	£0	£16,313	£0	£3,038	-
Costs incurred on false negatives	£8,369	£3,682	£7,771	£11,371	£14,721	-
Costs incurred on TST non-returns	£6,622	£0	£0	£6,622	£6,622	-

All costs are expressed in British pounds (£). Costs relate to outcomes and costs for entire cohort of 1,000 contacts over the 2 year examination period. (TST - Tuberculin Skin Test, QFT-GIT - Quantiferon-TB Gold-In-Tube)

Table 3 shows the effectiveness measures of each screening strategy. While the single IGRA screening strategies were the most costly, they were also the most effective at preventing cases of post-primary TB. Compared to conducting no screening, the T-SPOT.*TB *alone prevented 3.7 cases per 1000 contacts screened while the QFT-GIT prevented 3.47 cases per 1000 contacts screened. The dual strategies (TST/QFT-GIT and TST/T-SPOT.*TB*) resulted in the most post-primary TB cases occurring for the 2 year time frame (4.85 and 4.67 per 1000 contacts screened, respectively) and subsequently prevented the lowest number of TB cases (2.65 and 2.83 per 1000 contacts, respectively). The TST was the least effective of the single screening strategies (but more effective than the dual strategies), averting 2.98 cases of TB out of 1000 screened contacts. Another measure of effectiveness calculated was the number-needed-to-treat (NNT) i.e. the number of contacts treated for LTBI to prevent a case of active TB. Of the 1000 contacts screened using the TST alone strategy, 284 contacts were treated for LTBI resulting in an NNT of 95.5. This was the highest of all the strategies due to the large number of false positive that were given LTBI treatment (101 contacts). The T-SPOT.*TB *single and dual strategies had the lowest NNT of all the strategies (61.7 contacts). The QFT-GIT strategies were somewhat less efficient with an NNT of 69.7 and 63.6 for the single and dual strategies respectively.

**Table 3:** Analysis of effectiveness of each screening strategy

Effectiveness Measures	TST	T-SPOT.TB	QFT-GIT	TST/T-SPOT.TB	TST/QFT-GIT
Numbers of post-primary TB cases in 2 year period	4.52	3.80	4.03	4.67	4.85

Number of TB cases prevented by screening strategy	2.98	3.70	3.47	2.83	2.65

**Numbers of people treated for LTBI**	284	228	242	174	168
Numbers of true positives treated	184	228	214	174	163
Numbers of false positives treated	101	0	28	0	5

**Numbers of people treated for LTBI per case of active TB prevented (NNT)**	**95.5**	**61.7**	**69.7**	**61.7**	**63.6**

Effectiveness measures relate to outcomes and costs for entire cohort of 1,000 contacts over the 2 year examination period. No screening results in 7.5 cases of post-primary TB in the 2 year period. (TST - Tuberculin Skin Test, QFT-GIT - Quantiferon-TB Gold-In-Tube, NNT- number needed to treat)

Table 4 shows the cost effectiveness measures of each strategy. Cost effectiveness of each strategy was given as the incremental cost per active TB case prevented which represents the additional cost of a strategy over the baseline cost of not screening. TSPOT.*TB *dual screening was the most cost effective strategy costing £37,206 per TB case prevented followed closely by the QFT-GIT dual screening strategy (£37,699 per active TB case prevented). The IGRA single strategies were the next most cost effective options (T-SPOT.*TB *and QFT-GIT; £39,712 and £42,051 per case of active TB prevented, respectively). In both versions of the IGRA, dual screening was more cost effective than single screening; TST/T-SPOT.*TB *was £2,506 better than the T-SPOT.*TB *single strategy and TST/QFT-GIT was £4,351 better than screening with QFT-GIT only. T-SPOT.*TB *based strategies were also more cost effective than QFT-GIT based strategies. Screening with the TST alone remained the least cost effective strategy at £47, 840 incremental cost per case of active TB prevented.

**Table 4:** Analysis of cost-effectiveness of each screening strategy

Cost-effectiveness Measures	TST	T-SPOT.TB	QFT-GIT	TST/T-SPOT.TB	TST/QFT-GIT
Total costs of screening	£199,589	£203,983	£202,921	£162,387	£157,048

Incremental cost of screening (compared to no screening)	£142,442	£146,836	£145,774	£105,240	£99,901

Active TB cases prevented	2.98	3.70	3.47	2.83	2.65

Cost per active TB case prevented	£67,034	£55,168	£58,536	£57,410	£59,265

**Incremental cost per active case prevented (compared to no screening)**	**£47,840**	**£39,712**	**£42,051**	**£37,206**	**£37,699**

**Savings per active TB case prevented (compared to TST)**	**-**	**£8,128**	**£5,790**	**£10,634**	**£10,141**

All costs are expressed in British pounds (£). Cost-effectiveness relates to outcomes and costs for entire cohort of 1,000 contacts over the 2 year examination period. (TST - Tuberculin Skin Test, QFT-GIT - Quantiferon-TB Gold-In-Tube)

Different results were produced when total cost per active TB case prevented of each strategy was calculated based on the total costs of each strategy (rather than incremental costs). Using this measure, IGRA single strategies were actually found to be more cost effective than their dual strategy counterparts. The T-SPOT.*TB *alone was the most cost effective strategy costing £55,168 per active TB case prevented. The T-SPOT.*TB *dual strategy (£57,410 per active TB case prevented) was the next best from a cost effectiveness standpoint, followed by the QFT-GIT single and dual strategies (£58,536 and £59,265 per active TB cases prevented respectively). The TST alone was still the least cost effective option (£67,034 per active TB case prevented).

### Sensitivity analysis

Univariate deterministic sensitivity analysis identified a range of possible cost effectiveness outcomes, in terms of the incremental cost per case of active TB, for all variables (table 5 and 6). In most cases, when variables were changed within their specified ranges, T-SPOT.*TB *dual screening was the most cost effective strategy while screening with the TST alone was the least cost effective strategy.

**Table 5:** Cost-effectiveness of screening strategies when probabilities are varied in the sensitivity analysis.

	Incremental cost per Active TB case prevented (British Pounds; £'s)				
Probability Variable					
	TST	T-SPOT.*TB*	QFT-GIT	TST/T-SPOT.*TB*	TST/QFT-GIT
**Base-case estimates**	£47,840^5#^	£39,712^3^	£42,051^4^	£37,206^1^	£37,699^2^

**Prevalence**					
0.1	£109,120^5^	£69,462^3^	£80,160^4^	£55,118^1^	£58,321^2^
0.4	£40,180^5^	£35,994^3^	£37,287^4^	£34,967^1^	£35,122^2^

**T-SPOT.*TB*sensitivity**					
0.83	£47,840^5^	£41,863^3^	£42,051^4^	£38,994^2^	£37,699^1^
0.97	£47,840^5^	£39,406^3^	£42,051^4^	£36,951^1^	£37,699^2^

**T-SPOT.*TB*specificity**					
0.92	£47,840^5^	£46,090^4^	£42,051^3^	£38,707^2^	£37,699^1^
0.99	£47,840^5^	£40,510^3^	£42,051^4^	£37,394^1^	£37,699^2^

**QFT-GIT sensitivity**					
0.85	£47,840^5^	£39,712^3^	£42,862^4^	£37,206^1^	£38,305^2^
0.95	£47,840^5^	£39,712^3^	£40,962^4^	£37,206^2^	£36,887^1^

**QFT-GIT specificity**					
0.9	£47,840^5^	£39,712^3^	£46,302^4^	£37,206^1^	£38,701^2^
0.97	£47,840^5^	£39,712^3^	£40,350^4^	£37,206^1^	£37,299^2^

**TST sensitivity**					
0.69	£53,174^5^	£39,712^2^	£42,051^4^	£39,039^1^	£39,778^3^
0.95	£45,419^5^	£39,712^3^	£42,051^4^	£36,374^1^	£36,756^2^

**TST specificity**					
0.65	£61,205^5^	£39,712^2^	£42,051^4^	£39,044^1^	£40,055^3^
0.9	£38,931^3^	£39,712^4^	£42,051^5^	£35,981^1^	£36,129^2^

**Starting LTBI treatment**					
0.55	£50,196^5^	£46,474^3^	£47,951^4^	£42,828^1^	£43,091^2^
0.95	£47,022^5^	£37,364^3^	£40,001^4^	£35,253^1^	£35,827^2^

**Severe INH hepatitis**					
0.001	£47,653^5^	£39,576^3^	£41,923^4^	£37,073^1^	£37,563^2^
0.023	£49,743^5^	£41,100^3^	£43,341^4^	£38,559^1^	£39,082^2^

**Efficacy of 6 months INH treatment**					
0.5	£64,457^5^	£53,894^3^	£56,918^4^	£50,637^1^	£51,278^2^
0.93	£31,154^5^	£25,472^3^	£27,114^4^	£23,719^1^	£24,064^2^

**TST return rate**					
0.95	£47,680^5^	£39,712^3^	£42,051^4^	£37,037^1^	£37,519^2^
0.65	£49,012^5^	£39,712^3^	£42,051^4^	£38,439^1^	£39,016^2^

**Post exposure TB**					
0.01	£52,412^5^	£44,284^3^	£46,619^4^	£41,778^1^	£42,271^2^
0.05	£40,221^5^	£32,093^3^	£34,437^4^	£29,586^1^	£30,080^2^

^# ^The superscripts ^1-5 ^indicate the cost-effectiveness ranking; ^(1) ^indicates most cost effective strategy while ^(5) ^indicates the least cost-effective strategy. (LTBI - latent tuberculosis infection, TST - tuberculin skin test, INH - isoniazid, QFT-GIT - Quantiferon-TB Gold-In-Tube)

**Table 6:** Cost-effectiveness of screening strategies when cost estimates are varied in the sensitivity analysis.

	Incremental cost per Active TB case prevented (British Pounds; £'s)				
Cost Variable					
	TST	T-SPOT.*TB*	QFT-GIT	TST/T-SPOT.*TB*	TST/QFT-GIT
**Basecase Estimates**	£47,840^5#^	£39,712^3^	£42,051^4^	£37,206^1^	£37,699^2^

**TST cost**					
£8.07	£45,249^5^	£39,712^3^	£42,051^4^	£34,478^1^	£34,788^2^
£32.28	£53,024^5^	£39,712^1^	£42,051^2^	£42,662^3^	£43,523^4^

**T-SPOT.*TB *cost**					
£45.00	£47,840^5^	£37,008^2^	£42,051^4^	£35,949^1^	£37,699^3^
£100.00	£47,840^4^	£51,883^5^	£42,051^2^	£42,862^3^	£37,699^1^

**QFT-GIT cost**					
£35.00	£47,840^5^	£39,712^4^	£39,166^3^	£37,206^2^	£36,358^1^
£80.00	£47,840^4^	£39,712^2^	£52,147^5^	£37,206^1^	£42,395^3^

**Cost LTBI treatment**					
£262.30	£22,793^3^	£23,542^4^	£23,775^5^	£21,036^2^	£21,031^1^
£1,049.18	£97,937^5^	£72,053^3^	£78,603^4^	£69,547^1^	£71,038^2^

**INH hepatitis**					
£314.56	£47,750^5^	£39,654^3^	£41,985^4^	£37,148^1^	£37,639^2^
£1,258.24	£48,021^5^	£39,829^3^	£42,182^4^	£37,322^1^	£37,819^2^

**Post exposure TB**					
£3,809.84	£51,650^5^	£43,522^3^	£45,858^4^	£41,016^1^	£41,509^2^
£15,239.34	£40,221^5^	£32,093^3^	£34,437^4^	£29,586^1^	£30,080^2^

^# ^The superscripts ^1-5 ^indicate the cost-effectiveness ranking; ^(1) ^indicates most cost effective strategy while ^(5) ^indicates the least cost-effective strategy. (LTBI - latent tuberculosis infection, TST - Tuberculin Skin Test, INH - isoniazid, QFT-GIT - Quantiferon-TB Gold-In-Tube)

Changing LTBI prevalence had a significant impact on overall cost effectiveness. At the lower prevalence estimate (10%), cost effectiveness values significantly increased due to screening preventing less downstream cases of active TB (this is logical as, *in extremis*, screening a population with no infection is completely ineffective). Increase in prevalence (40%) resulted in each strategy becoming more cost effective compared to the base-case estimates due to the prevention of greater numbers of future active TB cases. However cost effectiveness (CE) rankings did not change within the parameters of the sensitivity analysis.

In our model, test sensitivities and specificities were varied according to the range of values reported in the literature. A lower test sensitivity (TST, QFT-GIT or T-SPOT.*TB*) reduces the cost of that strategy as fewer cases of true LTBI are detected and treated. However, these missed LTBI cases result in a greater number of post-primary TB cases for the 2 year screening period. As a result, lowering test sensitivity decreases the cost effectiveness of that strategy. CE rankings changed in favour of the QFT-GIT based strategies only if QFT-GIT sensitivity is ~8% higher than T-SPOT.*TB *sensitivity. Similarly, screening with TST only becomes more cost-effective than both IGRA test options (QFT-GIT or T-SPOT.*TB *single and dual) if the sensitivity of the TST is ~38% more than the sensitivity of T-SPOT.*TB *and ~40% greater than the sensitivity of QFT-GIT.

The strategy with a less specific test will cost more due to greater treatment and follow-up costs arising from false positive diagnoses without changing effectiveness measures in the model. Thus, lowering test specificity decreases the cost effectiveness of a particular strategy. Reducing T-SPOT.*TB *specificity to 92% makes the QFT-GIT dual strategy (£37,699 per active TB case prevented) more cost effective than the T-SPOT.*TB *dual strategy (£38,707 per active TB case prevented). The T-SPOT.*TB *single strategy became the least cost-effective when T-SPOT.*TB *specificity fell below 85%, all other things being equal. Lowering QFT-GIT specificity to the lower limit of the sensitivity analysis does not change the CE rankings. QFT-GIT-based strategies become more cost-effective than T-SPOT.*TB- *based strategies if QFT-GIT specificity is within ~1% of T-SPOT.*TB *specificity. TST only screening becomes more cost-effective than either IGRA single strategy if TST specificity is within ~6% of T-SPOT.*TB *or QFT-GIT specificity.

Greater efficacy of a 6 month course of INH improved the overall cost effectiveness of the screening strategies, as more persons are successfully cured of LTBI. If efficacy was increased to 93%, the CE ranking remained consistent but the incremental cost per active TB case prevented for each strategy decreased by approximately £15,000 per 1000 contacts screened compared to base-case estimates.

At lower TST return rates, dual (TST/QFT-GIT and TST/T-SPOT.*TB*) and TST alone strategy costs decrease as fewer LTBI individuals are identified and treated. However, more TST non-returners would progress to post primary TB in the two year time frame. As a result, cost effectiveness of these strategies is reduced. At 65% return rate, CE ranking remained consistent. However, T-SPOT.*TB *or QFT-GIT dual strategies became less cost effective than their single strategy counterparts when the TST return rate was ≤ 50% and ≤ 39%, respectively.

The incidence of post exposure TB did not significantly impact the cost effectiveness of the screening strategies. Different estimates of post exposure TB incidence not only changed overall costs and effectiveness measures of each strategy but also those of the 'no screening' scenario. As a result, the incremental cost per case of active TB prevented did not dramatically change. At a 1% incidence over 2 years, the incremental cost per case of active TB prevented increased by ~£5,000, compared to base-case estimates. The reverse occurred when the rate of post exposure TB was 5% i.e. incremental cost per active TB case prevented decreased by ~£5,000. The CE rankings remained consistent in each case.

Varying test costs and LTBI treatment costs had the greatest impact on cost effectiveness. When IGRA test costs were increased to the upper limit of the sensitivity analysis estimate, the gap in cost-effectiveness between IGRA single strategies and their dual strategy counterparts widened. However, all IGRA strategies (single and dual) still remained more cost effective than the TST alone option. Only when T-SPOT.*TB *and QFT-GIT costs were increased to ≥ £140 and ≥ £121 did the TST become the most cost effective option.

A 50% reduction in LTBI treatment costs reduced the incremental cost per case of active TB prevented for each strategy compared to the base-case analysis. The CE rankings also changed so that the TST became more cost effective than both IGRA single strategies. However the IGRA dual strategies remained the most cost effective. Doubling LTBI treatment costs almost doubled incremental cost per case of TB prevented for each strategy compared to the base-case analysis but CE ranking remained consistent.

## Discussion

Our analysis indicates that use of IGRAs as a screening tool, either alone or in conjunction with the TST, for detecting LTBI is a more cost effective alternative than using the TST alone. These results were consistent regardless of which IGRA (T-SPOT.*TB *or QFT-GIT) was used.

Dual screening, despite being less effective in terms of active cases prevented, was less costly and consequently more cost effective (in terms of incremental cost per TB case prevented) than IGRA single screening. The T-SPOT.*TB *dual strategy was actually found to be the most cost effective option (£37,206 per case of active TB prevented), followed very closely by the QFT-GIT dual strategy, which, while cheaper than the T-SPOT.*TB *dual strategy by £5,339, resulted in 0.18 more cases of active TB. The T-SPOT.*TB *single strategy prevented the most cases of post primary TB (3.70 cases) but was less cost-effective than the T-SPOT.*TB *dual strategy as it cost £41,596 more than the T-SPOT.*TB *dual strategy. Similar results were seen when QFT-GIT single and dual strategies were compared.

Screening with T-SPOT.*TB *was more cost effective than QFT-GIT screening when the dual (TST/QFT-GIT vs. TST/T-SPOT.*TB*) and single (QFT-GIT vs. T-SPOT.*TB*) strategies were directly compared. While the costs incurred due to false positive and false negative results were greater with the QFT-GIT based strategies (single and dual), the higher test cost of the T-SPOT.*TB *resulted in slightly higher overall costs of the T-SPOT.*TB *single or dual strategies. This was offset by higher effectiveness (fewer cases of post-exposure TB) resulting from the T-SPOT.*TB *strategies.

Screening with TST alone had the lowest testing costs (£15,433 per 1000 contacts), but incurred the highest costs due to test inaccuracies (£70,081). While this strategy prevented more cases of active TB than the dual strategies (but not the single IGRA strategies), it had the highest NNT (95.5) as over one third of the contacts treated for LTBI would be false positives. As a result, it was the least cost effective strategy.

Interpretation of the results will depend on the prevalence of infection, which within the UK and even within districts of London varies widely [25]. Prevalence in the screening group will also depend on the length of contact and immigration status (high-incidence or low-incidence), which may, in some part, explain why results of some studies were different to ours [10,16]. If prevalence is >52% then single testing with the T-SPOT.*TB *becomes more cost effective than dual testing. The same occurs with QFT-GIT screening when prevalence is >59%. At this prevalence the TST also becomes more cost effective than the dual strategies.

Overall our findings agree with recommendations by NICE [7] for a sequential screening strategy using the IGRA on all positive TSTs, despite different assumptions on LTBI prevalence and test performance estimates. The NICE cost-effectiveness analysis assumed that IGRAs had equal sensitivity and only slightly higher specificity than the TST, which may have reflected the relatively small number of publications available on IGRAs at the time this analysis was performed in 2004. Additionally, the NICE cost effectiveness analysis assumed a much lower prevalence of LTBI in contacts (8%). If we changed our estimates to equal those of the NICE analysis (lower prevalence of LTBI, equal test sensitivities), our findings still match those of the NICE analysis, that the dual strategy (compared to an IGRA single strategy) is the most cost-effective.

Other cost-effectiveness analyses investigating similar populations and examining effectiveness in terms of prevention of downstream active TB cases have drawn similar conclusions to our study. Diel *et al *[9] using similar test performance estimates to our analysis, compared similar strategies for the screening of close contacts using the QFT assay and found the dual strategy to be more cost-effective than the QFT single strategy. Oxlade *et al *[12] concluded from a study comparing TST and QFT in screening immigrants and contacts that a TST/QFT strategy is more cost effective than QFT single screening, particularly in a BCG vaccinated population.

Two studies have findings different to ours, supporting a single IGRA testing strategy ahead of a dual screening strategy. A Swiss study by Diel *et al *[10] found that T-SPOT.*TB *single screening was more cost effective than dual screening. However in this study cost effectiveness was determined using total costs rather than incremental costs. Using total cost outcomes from our study, we find similar results to Diel's study. Another Canadian study [16] found QFT screening to be more cost effective than TST/QFT screening in BCG vaccinated contacts when incremental net monetary benefit was compared. If the cost per TB case averted was compared then both strategies were found to be dominant. Additionally, unlike our study which compared each strategy to no screening, this study compared each strategy to screening with TST only.

A significant factor in determining the CE rankings of these strategies is the measure of cost effectiveness used. Dual screening was more cost effective than single screening when the incremental cost per case of active TB was used as the cost effectiveness measure. However, if total costs of a strategy rather than incremental costs were considered to calculate the cost per TB case prevented, single IGRA strategies were actually found to be more cost effective than dual strategies. Regardless of which measure is used, the TST only strategy remains the least cost effective strategy.

To our knowledge, there have been no direct cost comparisons between the two standardized versions of the IGRAs. UK estimates of QFT-GIT costs are generally lower than the T-SPOT.*TB *as the latter is more labour intensive and batch processing can greatly reduce QFT-GIT test costs. However T-SPOT.*TB *remains more cost-effective due to its seemingly superior test performance over the QFT-GIT [4].

Although they were not included in the model, we acknowledge that there are a number of other practical issues that must be taken into consideration when choosing a screening strategy (TST or IGRAs alone, or, as part of a dual strategy). There is some early evidence to suggest that using IGRAs for LTBI screening increases compliance to INH treatment [26]. In our model, better compliance would increase INH efficacy. Subsequently, the IGRA single and dual strategies would become even more cost effective.

Some studies have not accounted for non returns (assumed 100% TST return rate) [9,10,12,16] which in our model, accounts for £6,622 per 1000 contacts screened and results in 0.75 more cases of post primary TB. We assumed that there will be a stringent follow up of contacts thus we used a return rate of 90%. In reality, up to 60% of individuals fail to return for their TST results [27,28]). At these lower return rates, single IGRA strategies become more cost-effective than their dual strategy counterparts. Thus, IGRAs increase the proportion of individuals whose infection status is evaluable (LTBI or no LTBI) and saves time and money wasted on chasing up people to return for their TST to be read. Indeed, for this reason, the Health Protection Agency in the UK [29] recommends IGRA single testing in situations when screening large numbers of individuals makes testing with the TST problematic due to the large number of people that need to be followed up for TST reading. Nonetheless, it must be recognized that IGRAs also have some logistical problems. These include indeterminate results obtained (~5% to 15% of assays [30-32] in some studies), patients' refusal to have phlebotomy (8% in one recent study [28]), difficulty in obtaining blood from children (17% in a study in South Africa [33]), the need for additional phlebotomy services, limitations imposed by cut-off times for specimen transport, and patchy availability of these tests in the UK due to cost constraints.

Some studies did not include drug-related toxicity in their model [9,10,12]. Although isoniazid-associated hepatotoxicity is uncommon, when it does occur, it is difficult to treat and total costs are significant when large numbers of individuals are offered chemoprophylaxis. For example in the USA, where there are an estimated 350 000 treatment starts for LTBI annually (2002 estimate [34]), the total cost for treating isoniazid-associated hepatotoxicity may be over $1 million dollars per annum (assuming a hepatotoxicity risk of 0.3%). However when hepatitis rates were varied or even if excluded from this analysis, incremental costs per active TB case prevented only changed slightly but CE rankings remained consistent showing that the choice of testing strategy is insensitive to this variable.

The conclusion that is consistent in our study and previous cost analyses is that screening with the TST alone is not cost effective compared to strategies using the IGRA. However, whether IGRAs should be used as a replacement to the TST or in conjunction with it remains debatable. IGRA single screening does cost more (higher testing costs) but less money is spent on false negatives. Less false negatives means fewer people will progress to active disease, resulting in these strategies being the most effective (prevents most cases of post-primary TB). However the greater effectiveness of the single strategies does not overcome the lower cost of the dual strategies when incremental cost effectiveness is calculated.

Optimal choice of strategy will depend on logistical constraints as discussed above, and the population being screened as well as assumptions made in the costing model. For example, TST sensitivity is impaired in an immunocompromised population, such as HIV infected patients [35,36]. IGRA single screening strategies will undoubtedly be more suitable in this situation. Where the cost or clinical consequences of treating active TB are higher, such as with multi-drug resistant TB [37], then the benefit of identifying and treating people in the latent phase are even more pronounced, which may favour an IGRA single strategy.

There are several limitations of this study. Our analysis used a shorter timeframe of 2 years (other analyses used Markov modeling over a 20 year period [9,10,12,15,16]) and excluded wider transmission. We used this timeframe as Markov modeling would have added considerable complexity to the model. Additionally, healthcare institutions prefer to examine how implementation of a new clinical intervention affects their annual budget rather than long term overall costs over a 20 year period. However, a shorter timeframe underestimates the number of downstream active TB cases. Thus the more effective strategies in our model (T-SPOT.*TB *and QFT-GIT only) will become more cost effective if a longer timeframe was used. Similarly wider transmission underestimates the costs of less effective strategies. If included, single IGRA strategies may become more cost-effective as more future TB cases will be avoided. Nonetheless, it is reassuring that the results of our model in terms of ranking of the screening strategies are similar to analyses that have included Markov processes [9].

Health assessment agencies typically use Quality of Life Years (QALYs) as their outcome measure when conducting cost-effectiveness analyses. Our analysis did not include this measure due to limited UK data. Given that the main source of quality of life losses is active TB cases (as the risk of hepatitis is small) then strategies which prevented the most active TB cases may become more cost effective if QALYs were included. Additionally, we only included costs from the narrow perspective of the UK healthcare provider and did not include costs to the wider society i.e. costs incurred on patients, costs due to death, etc.

As there is no gold standard for detecting LTBI, data on IGRA sensitivity and specificity are typically taken from populations of active TB and healthy contacts, respectively. Changes in these parameters can change the CE rankings in our model. Consequently, more prospective UK data on the performance of these IGRAs is needed to better estimate these values.

Other LTBI treatment regimens (3 months isoniazid and rifampicin) have been recommended by the British Thoracic Society [19] as alternatives to the currently used 6 months of INH. Furthermore, other countries, such as the USA, recommend the use of either 9 months INH or 4 months of rifampicin for treating LTBI [38]. However these effects are complex to model. Different regimens will affect certain parameters in the model, including effectiveness and costs of LTBI treatment, compliance (shorter regimens will be tolerated better), risk of hepatotoxicity and other adverse drug effects. Modeling these different treatment regimens was beyond the scope of this study but would be a fruitful area of future research.

Lastly, this model was constructed primarily to examine the consequences of screening contacts, which may limit its applicability to other cohorts. Nonetheless, the sensitivity analysis allows the results to be generalisable across other cohorts, with changes in LTBI prevalence and the risk of post-primary TB being key variables.

## Conclusions

This study shows that, within the context of the assumptions made, the TST/IGRA dual strategy is cheaper than using T-SPOT.*TB*, or QFT-GIT or TST alone, for the screening of TB contacts. While the T-SPOT.*TB *and QFT-GIT alone prevent more cases of active TB, this does not overcome the lower cost of the dual strategies and consequently, using the measure of incremental cost per case of TB prevented, then dual strategies are more cost effective than their single IGRA strategy counterparts. However, these conclusions are dependent on the population being screened and on assumptions made on the test performance of the IGRAs compared to the TST. Nonetheless, using the IGRA, whether in a single or dual strategy, is always cheaper than using only the TST. This finding is very insensitive to changes in variables within reasonable parameters. These data should be considered when selecting a suitable strategy for screening LTBI.

## Competing interests

The authors declare that they have no competing interests.

## Authors' contributions

AP was involved with data acquisition, performing the cost analysis and drafting and critical review of the manuscript; RFM helped with the study design, data acquisition and manuscript drafting; HB, JH, GS, AZ and GR were all involved in study design, data acquisition, analysis and drafting of the manuscript; MB was involved in analysis of data and manuscript revision; KD supervised the overall study design, data acquisition, analysis, manuscript drafting and final submission. All authors contributed to the manuscript and gave approval for final submission.

## Pre-publication history

The pre-publication history for this paper can be accessed here:

http://www.biomedcentral.com/1471-2466/10/7/prepub

## Supplementary Material

Additional file 1 **Supplemental data**. This section gives a brief description of the structure of the decision tree model used in the cost analysis, and also further explains the choice of the probability, cost estimates and clinical effectiveness measures used in this analysis. This document is in Microsoft Word 2003 format. Click here for file
