# Supplementary material for: Different screening strategies (single or dual) for the diagnosis of suspected latent tuberculosis: a cost effectiveness analysis
Source: BMC Pulm Med. 2010 Feb 22;10:7. doi: 10.1186/1471-2466-10-7 (PMC2837635; doi:10.1186/1471-2466-10-7)
Supplement: Additional file 1 — Supplemental data. This section gives a brief description of the structure of the decision tree model used in the cost analysis, and also further explains the choice of the probability, cost estimates and clinical effectiveness measures used in this analysis. This document is in Microsoft Word 2003 format. [file 1471-2466-10-7-S1.DOC]

Supplement Data for:

# Different screening strategies (single or dual) for the diagnosis of suspected latent tuberculosis: a cost-effectiveness analysis

This section gives a brief description of the structure of the decision tree model used in the cost analysis (figures 1-3), and also further explains the choice of the probability and cost estimates (table 1) as well as the effectiveness measures used.

**Decision Tree Models**

Five different screening scenarios were investigated in this cost analysis: (1) TST alone, (2) the T-SPOT.*TB* assay alone, (3) TST followed by T-SPOT.*TB* assay when TST was positive, (4) Quantiferon-TB-Gold-In-Tube (QFT-GIT) alone, and (5) TST followed by QFT-GIT when TST was positive. As the T-SPOT.*TB* and QFT-GIT are different versions of the IFN-γ release assay (IGRA), the decision tree for each version of the assay are interchangeable. Hence the screening steps in scenarios (2) and (4) and scenarios (3) and (5) are identical.

When screening with the TST ([figure 1](http://mc.manuscriptcentral.com:80/LongRequest/erj?TAG_ACTION=DOWNLOAD_FILE_BY_NAME&DOCUMENT_ID=3850489&FILE_TO_DOWNLOAD=3850489_File000037_51728710.jpg.htm&FILE_KEY=-1574548475&FILE_NAME_KEY=1837595169&DOWNLOAD=TRUE&FILE_TYPE=DOCUMENT&DOCUMENT_HASHCODE=406531004&SANITY_CHECK_DOCUMENT_ID=3850489)), a contact may or may not return to have the test interpreted (‘TST read’ and ‘TST not read’). If the TST is not read then a contact may have LTBI which can remain latent (‘TB remains latent’) or progress to cause active TB (‘post-exposure TB’). For a contact returning for TST interpretation, a test result can be either positive or negative. For the T-SPOT.*TB* or QFT-GIT scenario ([figure 2](http://mc.manuscriptcentral.com:80/LongRequest/erj?TAG_ACTION=DOWNLOAD_FILE_BY_NAME&DOCUMENT_ID=3850489&FILE_TO_DOWNLOAD=3850489_File000038_51728722.jpg.htm&FILE_KEY=-1574548475&FILE_NAME_KEY=-716805279&DOWNLOAD=TRUE&FILE_TYPE=DOCUMENT&DOCUMENT_HASHCODE=406531004&SANITY_CHECK_DOCUMENT_ID=3850489)), the screening steps are identical to the TST decision tree except that, as there is no need for a return visit, all contacts would produce either a positive or negative test result. In the dual strategies ([figure 3](http://mc.manuscriptcentral.com:80/LongRequest/erj?TAG_ACTION=DOWNLOAD_FILE_BY_NAME&DOCUMENT_ID=3850489&FILE_TO_DOWNLOAD=3850489_File000039_51728938.jpg.htm&FILE_KEY=-1574548475&FILE_NAME_KEY=453089319&DOWNLOAD=TRUE&FILE_TYPE=DOCUMENT&DOCUMENT_HASHCODE=406531004&SANITY_CHECK_DOCUMENT_ID=3850489)), the T SPOT.*TB* or QFT-GIT is only performed on contacts with a positive TST. The positive predictive value (PPV) refers to the probability that a positive result is truly positive. Therefore a positive test can be truly positive (PPV) or falsely positive (1-PPV). Similarly, the negative predictive value (NPV) refers to the probability that a negative result is truly negative. Therefore a negative test can be a truly negative (NPV) or falsely negative (1-NPV).

**True Positive (PPV)**

A contact with a truly positive test result is assumed to have LTBI and can either accept (‘start INH treatment’) or refuse isoniazid treatment (‘no INH treatment’). If treatment is refused, TB can remain latent or progress to active TB. In a contact receiving INH treatment, there is a risk of severe isoniazid-induced hepatotoxicity (‘severe hepatitis’), which can result in death (‘death due to hepatitis’), or resolution of the hepatitis (‘hepatitis resolved’). If the hepatitis is resolved then the LTBI treatment can be successful and completely eliminate the disease (‘LTBI fully cured’) or unsuccessful allowing the TB to remain uncured (‘LTBI not cured’). If not cured, the TB can remain latent or can reactivate into active TB.

**False Positive (1-PPV)**

It is assumed that a person with a falsely positive result is not infected with latent TB. However, in the model, they will still be offered isoniazid treatment. If treated, there is still a risk of developing hepatitis which can be resolved or cause death. If treatment is refused, there is no further consequence. It is assumed that those who give a falsely positive test result undergo the same length of treatment as those with a truly positive result. Therefore, ‘hepatitis resolved’ is associated with the cost of partial course (3 months) of isoniazid treatment, whereas ‘no hepatitis’ is associated with the cost of a full course (6 months) of isoniazid treatment.

**True Negative (NPV)**

It is assumed that a contact with a negative test result has no further investigation. A

contact that has a truly negative result is free of infection with no further consequence.

**False negative (1-NPV)**

No treatment is offered for a falsely negative result. However, it is assumed the person is latently infected. As a result, the TB can remain latent or can progress to active TB

**Probabilities**

All probability estimates were taken from the published literature.

**LTBI prevalence**

Estimates of LTBI prevalence can vary widely depending on the study population and the duration of exposure to the index case. Previous studies have shown an estimated 30 – 40% [1, 2] prevalence of LTBI in contact tracing studies conducted in the USA and the UK. A baseline estimate of 30% was used and a wider range was examined in the sensitivity analysis in order to cover different contact tracing scenarios.

Bayesian statistics were used to calculate the probability of a positive and negative test result from LTBI prevalence and test specificity and sensitivity:

**Probability of a positive result = (Sensitivity * Prevalence) + ((1 - Specificity) * (1 - Prevalence))**

**Probability of a negative result = (Specificity * (1 - Prevalence)) + ((1 - Sensitivity) * Prevalence)**

Furthermore, positive predictive value and negative predictive values for each test were subsequently calculated:

**Positive Predictive Value = Sensitivity * Prevalence / Probability of a positive result**

**Negative Predictive Value = Specificity * (1 - Prevalence) / Probability of a negative result**

In the dual screening strategies, the prevalence of LTBI in the TST positive cohort will be higher than the background prevalence. As such, the probability of a positive and negative result, as well as the PPV and NPV, of the IGRA given a positive TST is different than these estimates in the IGRA only strategies. These values are shown in table 1.

**TST return rate**

Reported TST return rates were available mainly from U.S. studies on populations of drug users, ranging from 47% to 93% [3, 4]. In the UK, Bothamley reported a 73% return rate among a population of newly screened immigrants [5]. We assumed a 90% rate of return for TST readings (as contacts are more likely to be followed up), similar to previous cost analyses [6, 7], with lower rates explored in the sensitivity analysis.

**Test sensitivity and specificity**

There is no gold standard test for LTBI diagnosis. Therefore, as in most previous studies, sensitivity was obtained from a population of confirmed TB cases and specificity was obtained from a population of healthy contacts at low risk for exposure. T-SPOT.*TB* and QFT-GIT sensitivity and specificity values can vary depending on the screening population and study methods. In order to best approximate our UK study population, estimates of IGRA sensitivity and specificity were used from studies conducted in the UK or with similar population dynamics to the UK (low TB burden, moderate to high levels of immigration, BCG vaccination). Furthermore, T-SPOT.*TB* estimates were taken from IGRA studies using the ELISPOT platform [8-11] while QFT-GIT estimates were taken from studies using ELISA versions of the assay [12, 13]. Additionally, estimates from studies on immunosuppressed populations and those using other versions of the IGRA (prolonged incubation times, use of protein antigens) were omitted.

Sensitivity values for the T-SPOT.*TB* ranged from 83% to 97% [8-11]. In two UK studies, T-SPOT.*TB* sensitivities were 92% and 96% [9, 11]. A sensitivity of 95% was used for the base-case estimate. Specificity of the T-SPOT.*TB* ranges from 92%-100% with 2 UK studies [9, 11] and one German study[14] reporting 100%. Thus a base-case value of 100% was chosen for this analysis. Reported sensitivity estimates of the QFT-GIT were lower than the T-SPOT.*TB* [15], with values of 85% and 89% in two studies [12, 13]. We used the higher sensitivity value of 89% in the base-case analysis. Similarly, reported specificity of the QFT-GIT is slightly lower than in the T-SPOT.*TB*. Two studies report values of 97% and 90% [12, 13]. We used a base-case estimate of 95% in our analysis. Sensitivity of the TST was shown to be the same as or lower than that of the IGRA assays in similar populations, with values ranging from 69%-90% [8-10, 16]. A higher estimate of 85% was used as the base-case estimate. TST specificity also tends to be lower especially in BCG vaccinated populations. Reported figures fall between 79%-88% [17, 18]. We assumed a high degree of BCG vaccination therefore a baseline value of 80% was used for this analysis. High and low estimates for these parameters were explored in the sensitivity analysis.

**Isoniazid-related hepatitis**

The incidence of isoniazid-induced hepatitis is highly age dependent with persons > 50 years of age exhibiting higher rates of 2.3% [19]. Furthermore, while older studies have reported rates as high as 4.1% [20], more recent studies show rates tend to be lower once proper patient selection and treatment guidelines are followed. Two meta-analyses reported rates of 0.6% [21] and 0.36% [22]. A rate of 0.3% was used in our analysis but higher and lower rates were explored in the sensitivity analysis.

**Death from isoniazid-related hepatitis**

Salpeter reported an average rate of isoniazid hepatitis-related death of 0.01% from studies where anti-TB treatment was not monitored [23]. However, if current treatment monitoring guidelines are followed, unlike in the older studies, then much lower rates of 0.001% to 0.003% are found [23]. For this analysis, an isoniazid-hepatitis fatality rate of 0.002% was assumed but ranges were examined in sensitivity analyses.

**Efficacy of Isoniazid**

The probability of being fully cured is based on the efficacy of either a complete or incomplete course of isoniazid. The literature quotes estimates of isoniazid effectiveness ranging from 50% to 93% based on treatment durations of 6 months to one year [22, 24-29]. The IUAT study, a large randomized-controlled trial which investigated isoniazid treatment duration, reported an effectiveness of 65% for a 6 month course of isoniazid treatment (effectiveness of a 12 month course was 75%). [24]. These estimates assumed heterogeneity in the adherence and completion rates of those taking INH treatment. Estimates were higher in those who complied to and completed treatment [30] As such, 65% was used as the baseline value for 6 month INH efficacy in this model and the full reported range was investigated in the sensitivity analysis. Ranges for the effectiveness of a 3 month isoniazid course was reported to be between 12 and 30% [24, 27]. The IUAT trial reported 21-31% effectiveness for a 3 month period of INH [24].We therefore used a value of 21% for the efficacy of an incomplete course (3 months) and explored the ranges in the sensitivity analysis.

**Post exposure TB**

There is wide variation in the reported risk of post exposure TB mainly due to differences in the study population and length of time since the initial infection. It is widely accepted that the greatest risk of progression occurs immediately following initial infection and this risk drops as time passes. An estimated 50% of LTBI cases that may progress to active disease do so within two years of primary infection [6]. As this model focuses on the first 2 years after infection, a rate of 2.5% was used as the base-case, as in previous cost analyses [6, 31]. Higher and lower estimates from the published data were examined in the sensitivity analysis.

**Costs**

Unit costs were taken either from UK national sources or from published studies using the most available current estimates (table 1). All costs were updated to 2008 GPB using the Bank of England Consumer Price Index [32]. There was no time discounting of future costs as the time period of the model was only 2 years.

**IGRA**

The Royal Free Hospital in London estimated the cost of performing the T-SPOT.*TB* at £50. The reported cost of performing the QFT-GIT was £40 (estimate from Royal Blackburn Hospital). These costs include sample processing and performing of the test itself. Cost of phlebotomy was taken from national sources [33] and estimated to be £5. The cost of performing the IGRA is more expensive in private laboratories, with some laboratories charging £100 per test. As a result we have included a wide range of costs in the sensitivity analysis.

**TST**

Costs associated with the TST (either Heaf or Mantoux) were taken from the National Institute of Health and Clinical Excellence (NICE) [34]. TST cost components from the NICE analysis were updated to current estimates [32, 35].

**LTBI Treatment**

Estimates for a 6 month course of isoniazid were taken from NICE and included the cost of 6 months of isoniazid, chest X-rays, culture tests and clinic visits [34]. We calculated the cost of a 3 month isoniazid course by incorporating the cost of a 3 month supply of isoniazid course rather than a 6 month supply. Halving and doubling of the total treatment cost was used as the higher and lower estimates in the sensitivity analysis.

**Treatment for severe INH hepatotoxicity**

According to Schwartzmann and Menzies [18], the treatment of INH related hepatotoxicity requires 2 additional clinic visits and 2 full blood counts and biochemisty panels. Additionally, up to 20% of individuals require hospitalization for one week. The cost of the hospital stay, clinic visits and blood tests were taken directly from NHS sources [33]. Halving and doubling of the total treatment cost was used as the higher and lower estimates in the sensitivity analysis.

**Active TB Treatment**

This refers to the cost associated with treating a case of post-exposure TB. White and Moore-Gillion estimated the overall costs of treating a case of active TB in the UK, which was updated to 2008 costs [36]. Despite the costs of active TB being incurred in the second year after initial infection, we chose not to discount these costs back to 2008 GBP as the rate of inflation used by the consumer price index (3.9%) is similar the rate of discounting typically used in cost analyses (3.5%). Halving and doubling of the cost variables was used as the higher and lower estimates in the sensitivity analysis.

**Effectiveness measures**

The number of active TB cases prevented by each screening strategy was calculated by subtracting the number of active cases arising from a ‘no screening’ scenario from the cases arising from each screening strategy in the two year time-frame of the model. Additionally, the number needed to treat (NNT) which is the number of contacts that undergo LTBI treatment to prevent a case of active TB was calculated (table 3).

**Cost-effectiveness**

The primary measure of cost effectiveness used in our analysis was the incremental cost per active TB case prevented. This was calculated using incremental costs, defined as the additional costs of a particular strategy over and above the costs spent on the ‘no screening’ scenario (£57,148**)**, divided by the number of cases of active TB prevented by that strategy.

i.e. **Incremental cost per Cost of screening strategy – Cost of no screening**

**active TB case prevented** = ___________________________________________

**Active TB cases prevented by screening strategy**

Cost-effectiveness was also measured as the total cost per case of active TB prevented and was calculated using the total cost of each strategy divided by the number of active TB cases prevented by that strategy. The savings associated with each strategy when compared with the TST only screening strategy was also reported (Table 4).
